# Hypoperfusion-related functional abnormalities of middle cerebral artery stenotic-occlusive disease

**DOI:** 10.1093/braincomms/fcaf393

**Published:** 2025-10-14

**Authors:** Yinxi Zou, Anqi Cheng, Qianqian Si, Linwen Liu, Huanyu Zhou, Xiaoyuan Fan, Xiaoqian Zhang, Yiyang Liu, Ningyuan Liu, Haoyao Guo, Mingli Li, Caiyan Liu, Weihai Xu

**Affiliations:** Department of Neurology, State Key Laboratory of Complex Severe and Rare Diseases, Peking Union Medical College Hospital, Chinese Academy of Medical Sciences and Peking Union Medical College, Beijing 100730, China; Department of Neurology, State Key Laboratory of Complex Severe and Rare Diseases, Peking Union Medical College Hospital, Chinese Academy of Medical Sciences and Peking Union Medical College, Beijing 100730, China; Department of Neurology, State Key Laboratory of Complex Severe and Rare Diseases, Peking Union Medical College Hospital, Chinese Academy of Medical Sciences and Peking Union Medical College, Beijing 100730, China; Department of Neurology, The First Affiliated Hospital of Nanjing Medical University, Nanjing 210029, China; Theranostics and Translational Research Center, National Infrastructures for Translational Medicine, Institute of Clinical Medicine, Peking Union Medical College Hospital, Chinese Academy of Medical Sciences and Peking Union Medical College, Beijing 100730, China; Department of Neurology, State Key Laboratory of Complex Severe and Rare Diseases, Peking Union Medical College Hospital, Chinese Academy of Medical Sciences and Peking Union Medical College, Beijing 100730, China; Department of Radiology, State Key Laboratory of Complex Severe and Rare Diseases, Peking Union Medical College Hospital, Chinese Academy of Medical Sciences and Peking Union Medical College, Beijing 100730, China; Department of Radiology, State Key Laboratory of Complex Severe and Rare Diseases, Peking Union Medical College Hospital, Chinese Academy of Medical Sciences and Peking Union Medical College, Beijing 100730, China; Department of Neurology, State Key Laboratory of Complex Severe and Rare Diseases, Peking Union Medical College Hospital, Chinese Academy of Medical Sciences and Peking Union Medical College, Beijing 100730, China; Department of Neurology, State Key Laboratory of Complex Severe and Rare Diseases, Peking Union Medical College Hospital, Chinese Academy of Medical Sciences and Peking Union Medical College, Beijing 100730, China; Department of Neurology, State Key Laboratory of Complex Severe and Rare Diseases, Peking Union Medical College Hospital, Chinese Academy of Medical Sciences and Peking Union Medical College, Beijing 100730, China; Department of Radiology, State Key Laboratory of Complex Severe and Rare Diseases, Peking Union Medical College Hospital, Chinese Academy of Medical Sciences and Peking Union Medical College, Beijing 100730, China; Department of Neurology, State Key Laboratory of Complex Severe and Rare Diseases, Peking Union Medical College Hospital, Chinese Academy of Medical Sciences and Peking Union Medical College, Beijing 100730, China; Department of Neurology, State Key Laboratory of Complex Severe and Rare Diseases, Peking Union Medical College Hospital, Chinese Academy of Medical Sciences and Peking Union Medical College, Beijing 100730, China

**Keywords:** arterial spin labelling, cerebral hypoperfusion, cognitive impairment, functional connectivity, middle cerebral artery stenosis/occlusion

## Abstract

Unilateral asymptomatic middle cerebral artery stenosis or occlusion (MCAs/o) is an ideal human model for investigating the neural consequences of chronic cerebral hypoperfusion. Using a discovery-validation approach, this study aimed to characterize functional abnormalities in hypoperfused brain regions of unilateral MCAs/o and assess their neurobehavioral implications. In a discovery cohort comprising 41 patients with unilateral MCAs/o and 30 matched controls, patients exhibited significantly impaired performance on bilateral grooved pegboard tests (GPT, *P* < 0.05). Arterial spin labelling identified hypoperfused regions with prolonged arterial transit time. These regions showed increased intraregional regional homogeneity and functional connectivity (FC), and decreased extraregional FC (FDR-*P* < 0.05). A machine-learning model integrated these functional imaging features into a hypoperfusion-functional abnormality index (HFAi), which effectively detected early functional abnormalities in MCAs/o patients (AUC = 0.978) and correlated significantly with GPT performance (*P* < 0.01). Validation in an independent cohort (20 MCAs/o patients and 18 controls) confirmed these findings, demonstrating consistent identification of early functional abnormalities (AUC = 0.861) and correlation between HFAi and GPT scores (*P* < 0.05). Our results indicate that unilateral MCAs/o increased local neural synchronization coupled with reduced global functional integration, suggesting a shift towards isolated neural processing. These hypoperfusion-related functional abnormalities are closely linked to neurobehavioral alterations and can be objectively quantified.

## Teaser

Zou *et al*. report that in unilateral asymptomatic middle cerebral artery stenosis–occlusion, chronically under-perfused regions show stronger local synchrony but weaker long-range connectivity. They develop and validate a hypoperfusion-functional abnormality index quantifying these changes, which relates to individual cognitive differences, highlighting a reproducible marker for personalized risk assessment.

## Graphical Abstract

**Graphical Abstract:**
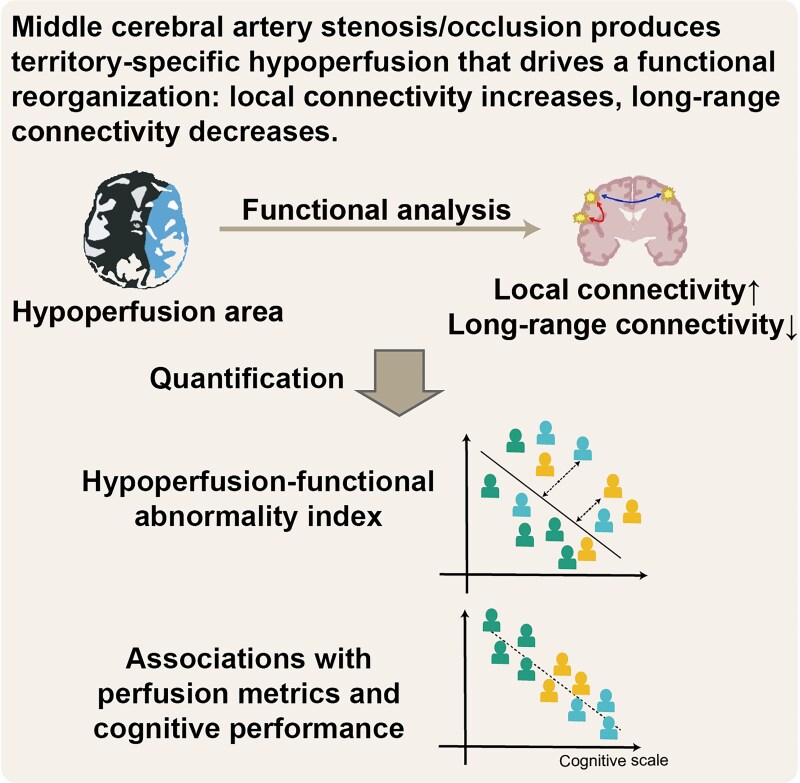

## Introduction

Chronic cerebral hypoperfusion is detrimental to brain health,^1-3^ which is associated with cognitive impairment and brain atrophy.^4-7^ As a principal contributor to chronic hypoperfusion, intracranial artery stenosis (ICAS) demonstrates particular clinical relevance due to its high prevalence in the general population.^8^ The Atherosclerosis Risk in Communities study has suggested that ICAS is associated with incidental dementia, independent of traditional vascular risk factors.^9^ The underlying mechanisms remain to be elucidated. Patients with ICAS do not have cerebral β-amyloid deposition, a hallmark of Alzheimer's disease, on PET imaging.^10^ In individuals with a low burden of white matter hyperintensities and without a history of ischemic stroke, ICAS was still associated with dementia.^9,11^ These observations collectively implicate chronic hypoperfusion as a potential pathophysiological pathway in causing brain dysfunction, warranting further mechanistic investigation.

Unilateral middle cerebral artery stenotic-occlusive disease (MCAs/o) provides an ideal natural human model of chronic cerebral hypoperfusion, offering clearly demarcated hypoperfusion territories (stenotic hemisphere) with minimal confounding from collateral circulation or bilateral compensatory mechanisms.^12,13^ Preliminary studies have shown that MCAs/o impairs cerebrovascular reserve,^14^ lead to reduced neuronal viability and metabolic dysregulation within the hypoperfused region (HypoR),^15^ and induces microstructural damage within the affected hemisphere.^16^ Our previous prior work further revealed that patients with asymptomatic unilateral MCAs/o exhibit extensive alterations in the functional network.^17^ However, the functional connectomic signature of chronic hypoperfusion in this population remains unexplored. It is an important knowledge gap, given that early neural network reorganization often precedes overt cognitive impairment. The absence of quantitative biomarkers to track these changes hinders our ability to differentiate adaptive plasticity from pathological dysfunction, ultimately limiting both mechanistic insights and therapeutic advancements.^18,19^

In this study, using a discovery-validation research pipeline in patients with asymptomatic unilateral MCAs/o, we sought to characterize perfusion-dependent neural dysfunction and its behavioural correlates in asymptomatic unilateral MCAs/o patients. Additionally, we explored to develop a machine learning-based biomarker to quantify functional abnormalities, leveraging its capacity for high-dimensional pattern recognition to enhance diagnostic precision and mechanistic understanding.

## Materials and methods

### Study design

**Figure 1:**
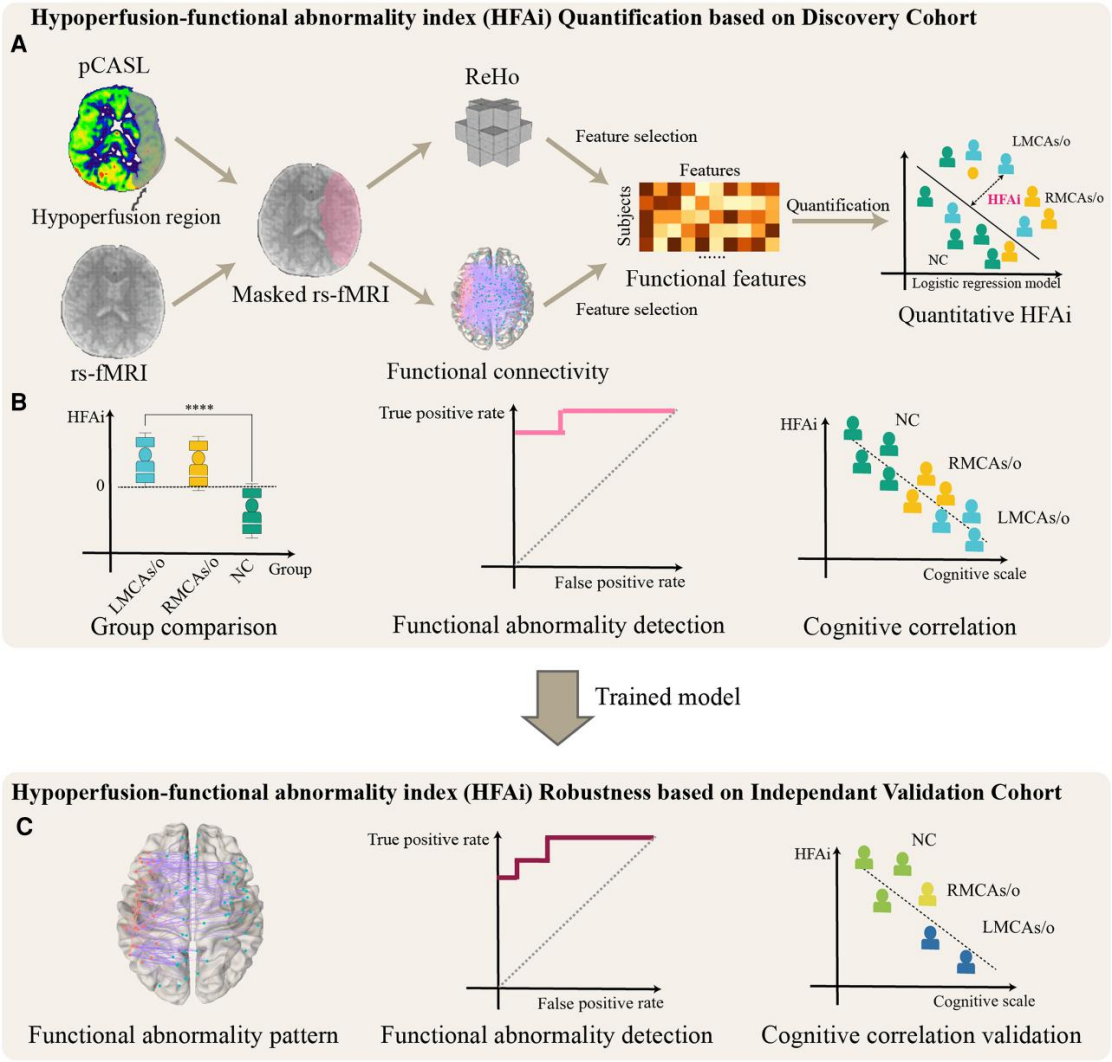
**Qualitative–quantitative validation research framework of this study.** (**A**) HFAi quantification based on discovery cohort: Pseudocontinuous arterial spin labelling imaging was first used to identify HypoR in the discovery cohort. Functional changes within HypoR, including ReHo and FC, were compared between groups and extracted as functional features for quantification. HFAi was then quantified using functional features based on machine learning methods; (**B**) inter-group comparison and correlation analysis with cognitive performance of HFAi; (**C**) HFAi robustness based on independent validation cohort. Note. MCAs/o, asymptomatic middle cerebral artery stenotic-occlusive disease; pCASL, pseudo-continuous arterial spin labelling MRI; rs-fMRI, resting-state functional MRI; ReHo, regional homogeneity; FC, functional connectivity; HFAi, hypoperfusion-functional abnormality index; NC, normal controls.

As shown in Fig. 1, to identify and quantify hypoperfusion-induced brain functional abnormalities in MCAs/o patients, we developed a qualitative–quantitative validation research framework combining advanced perfusion mapping and functional connectivity (FC) analysis which builds upon established computational psychiatry methodologies^20-22^: (i) We first identify hypoperfusion regions using pseudocontinuous arterial spin labelling imaging, followed by group comparisons of regional homogeneity (ReHo) and FC within these hypoperfused areas based on resting-state functional magnetic resonance imaging (rs-fMRI) to preliminarily and qualitatively assess the functional abnormalities. We then quantitatively calculate hypoperfusion-functional abnormality index (HFAi) using logistic regression models based on selected functional features, and separately analyse the correlations between perfusion metrics, cognitive performance and HFAi; (ii) finally, we validate the robustness of the HFAi findings by comparing the results between the discovery cohort and an independent validation cohort, ensuring the consistency and reliability of our conclusions.

### Discovery cohort

Consecutive patients with incidental unilateral MCA steno-occlusive atherosclerotic disease and without a history of stroke or transient ischemic attack were recruited from 1 January 2022 to 1 June 2024. MCA steno-occlusive disease was defined as >70% stenosis on maximum intensity projection images or a complete signal loss of the MCA trunk on magnetic resonance angiography.^23,24^ The asymptomatic status was confirmed through a detailed patient history review and a complete neurologic examination. Patients were excluded if they were: (i) over 85 years of age; (ii) referred or documented any other diseases or psychiatric diseases contributing to dementia, such as neuro-syphilis, HIV and depression; (iii) with extracranial carotid artery stenosis or occlusion diagnosed by carotid duplex or CT angiography and (iv) with bilateral MCAs/o or severe multiple ICAS. Normal controls (NC) were enrolled with matched age, education level and without any of the following: (i) complaint of memory function decline; (ii) existence of anxiety and/or depression; (iii) >50% stenosis of extra- and intracranial carotid artery and (iv) other diseases which may cause cognitive dysfunction.

### Independent validation cohort

To validate the stability of the functional abnormalities found in our study, we used an independent cohort from Peking Union Medical College Hospital, which included MCAs/o patients and NC. The inclusion and exclusion criteria for the independent validation cohort were the same as those for the discovery cohort. All patients in the independent validation cohort also underwent detailed neuropsychological cognitive testing and multimodal MRI examinations.

The ethics committee of the Peking Union Medical College Hospital approved this study (I-24PJ0461). All subjects or their relatives signed a written consent to participate.

### Neuropsychological assessment

All subjects underwent a detailed neuropsychological assessment. General intellectual function was assessed using the Mini-Mental State Examination,^25^ Montreal cognitive assessment (MoCA) (Chinese version)^26^ and a battery of neuropsychological tests including backward and forward digit span, paired associate word learning, Rey complex figure copy and recall test and the grooved pegboard test (GPT) using the dominant and non-dominant hand.

### Image acquisition

Multimodal MR imaging of the discovery cohort was performed on 3T Discovery systems (GE Healthcare) equipped with a 20-channel head coil. The scanning sequences included pseudocontinuous arterial spin labelling imaging, 3D T1-weighted brain structural imaging and rs-fMRI. All subjects of independent validation cohort underwent comprehensive neuropsychological assessments and multimodal MRI examinations, including 3D T1 and rs-fMRI scans. To test the generalizability of the model, these multimodal MRI scans were conducted on a MAGNETOM Skyra 3T MR scanner (Siemens, Erlangen, Germany). Despite the difference in scanner models, the scanning parameters for 3D T1-weighted imaging and resting-state functional MRI (rs-fMRI) sequences were identical between the two cohorts (Neuroimaging protocol detailed in Data S1).

### Image preprocess

#### Arterial serial labelling preprocess and determination of hypoperfusion areas

The quantitative data of subjects in the discovery cohort were automatically analysed using CereFlow software by AnImage (Beijing) Technology Co., Ltd. with these steps: (i) cerebral blood flow (CBF) and arterial transit time (ATT) data from raw 7-delay ASL data.^27^ (ii) Coregister M0 and T1 image and transform M0/ASL data to T1 space (3d rigid transformation); (iii) Coregister T1 and MNI152 template and transform connectional architecture-based brain atlas (Brainnetome Atlas, BNA)^28^ to T1 space (3d non-rigid transformation); and (iv) calculate mean CBF/ATT value of each delay and each BNA region of interest (ROI).

To identify the chronic hypoperfusion areas in patients with MCAs/o, we compared the perfusion indices based on BNA between MCAs/o patients and NC. ATT is more sensitive than CBF in pseudocontinuous arterial spin labelling imaging analysis of asymptomatic cerebral artery stenosis,^29^ ROIs with significantly different ATT were selected as hypoperfusion areas for further analysis in resting-state functional MRI.

#### Resting-state functional MRI preprocess

The T1-weighted and resting-state fMRI (rs-fMRI) data from discovery and validation cohorts from two MRI scanners were independently preprocessed using SPM12 (http://www.fil.ion.ucl.ac.uk/spm) and AFNI.^30^ Preprocessing steps for T1-weighted images included intensity non-uniformity correction, skull stripping, tissue segmentation and normalization via the DARTEL algorithm.^31^ For rs-fMRI, preprocessing included slice-time and motion correction, boundary-based registration to T1-weighted images and exclusion of participants with head motion >2.5 mm/2.5°. Confound regression removed 24 head motion parameters, white matter, cerebrospinal fluid signals and linear/quadratic trends. ReHo analysis, conducted with AFNI's 3dReHo (nneigh = 27), quantified local connectivity of low-frequency BOLD signals (0.01–0.08 Hz), including only voxels within individual masks. ReHo maps were smoothed with a 6 mm FWHM heat kernel. FC matrices were generated by calculating Pearson correlations between the mean time series of brain regions defined by BNA, with each matrix element representing the connection strength between two ROIs. The calculation of average FC for major functional networks was based on the definition of ROI functional network attributes according to the BNA.^28^

### HFAi calculation

We used a data-driven HFAi to quantify the FC abnormalities under chronic hypoperfusion conditions. Classification models can provide the confidence level of a patient's diagnostic status and are widely used to quantify abnormalities in patients’ imaging biomarkers.^21,32^ In our framework, HFAi is defined as the signed distance from each sample to the logistic regression decision boundary, providing a continuous, directionally interpretable index of functional abnormality. Positive values indicate a shift towards the MCAs/o pattern, negative values indicate a control-like pattern and a value of zero lies on the decision boundary.

Candidate features included FC between ROIs within the HypoR and between hypoperfused and non-hypoperfused ROIs, as well as ReHo within the HypoR. All features were z-standardized prior to modelling to place them on a comparable scale. Feature selection was performed using LASSO to obtain a sparse, informative feature set. The selected features were then used to train a logistic regression classifier. Model performance was estimated using 5-fold cross-validation in the discovery cohort, and the trained classifier was subsequently applied to the validation cohort to compute per-subject HFAi and assess robustness and generalizability. All analyses were implemented in scikit-learn.^33^

### Statistical analysis

Demographic data were summarized as mean ± SD, median [IQR], or percentages. ANOVA and chi-square tests assessed continuous and categorical variables, respectively. General linear regression analysed neuropsychological tests and MCAs/o, adjusted for age, gender and education. Two-sample *t*-tests compared FC and ReHo between groups, with FDR correction (MATLAB 2016b). Correlations with cognitive tests were analysed using Pearson correlation. Statistical analyses were performed using SPSS 20.0, R (version 4.0.5) and MATLAB, with significance set at *P* < 0.05.

## Results

Fifty-seven patients with MCA s/o were considered for enrolment in the discovery cohort. Four patients with combined intracranial carotid artery stenosis and eight with bilateral MCA s/o were excluded. Four participants with significant head motion during the rs-fMRI scans were also excluded. Finally, 41 patients were recruited, including 21 patients with left MCA s/o and 20 patients with right MCA s/o. Thirty NC subjects were enrolled with matched ages and education levels. Except for hypertension (*P* < 0.001) and hypercholesterolemia (*P* < 0.001), there were no significant differences in characteristics such as gender, age, years of education and risk factors for cardiovascular and cerebrovascular diseases amongst the three groups (Table 1). No significant differences were observed in the lacunar numbers, white matter hyperintensities and degree of brain atrophy (Supplementary Table 1).

**Table 1:** Demographic characteristic and neuropsychological assessment performance of the patients and controls in discovery cohort

	LMCAs/o patients (*n* = 21)	RMCAs/o patients (*n* = 20)	Normal control (*n* = 30)	*P*-value
**Age (year) (mean** **±** **SD)**	56.81 ± 10.88	49.25 ± 14.06	52.83 ± 11.24	0.14
**Male, No. (%)**	12 (57.1%)	15 (75%)	14 (46.7%)	0.14
**Education level (years, mean** **±** **SD)**	12 ± 4.14	13.35 ± 4.23	13.2 ± 5.13	0.58
**Hypertension, No. (%)**	12 (57.1%)	14 (70%)	6 (20%)	<0.001
**Diabetes, No. (%)**	5 (23.8%)	3 (15%)	2 (6.7%)	0.22
**Hypercholesterolemia, No. (%)**	19 (90.5%)	19 (95%)	3 (10%)	<0.001
**Atrial fibrillation, No. (%)**	0 (0.0%)	1 (5%)	0 (0.0%)	0.27
**Smoking, No. (%)**	7 (33.3%)	8 (40%)	7 (23.3%)	0.44
**MMSE (median [IQR])**	28[27–29]	29[28–30]	29[28–30]	0.20^a^
**MOCA (median [IQR])**	26[23–28]	28[26.5–29]	29[28–30]	0.006^a^
**Paired associate word learning test (median [IQR])**	12[7.5–16.5]	14[10.38–18]	14[9–17.88]	0.94^a^
**Rey complex figure copy (median [IQR])**	35[33–36]	36[34–36]	36[36–36]	0.09^a^
**Rey complex figure recall (median [IQR])**	19.5[16.5–23.5]	24.5[19.25–31.5]	26[19–29]	0.17^a^
**Grooved pegboard (dominant hand, median [IQR])**	72.34[62.67–77.14]	56.73[54.79–62.2]	59.53[52.46–65.47]	0.039^a^
**Grooved pegboard (non-dominant hand, median [IQR])**	75.61[67.35–87.24]	62.48[58.08–68.54]	60.58[54.48–70.42]	0.005^a^

^a^The general linear regression model was used to analyse the relationship between the neuropsychological test results and the MCAs/o adjusted by age, gender and education years.

After adjusting for age, gender and education years, there was a significant difference in MoCA (*P* = 0.006), dominant hand GPT (*P* = 0.039) and non-dominant hand GPT (*P* = 0.005) amongst the three groups. No significant difference in other neuropsychological test performance was found (Table 1). The results showed that MCAs/o patients experienced predominant declines in fine motor function, which was more pronounced in left MCAs/o patients.

### Analysis of functional abnormalities based on HypoR

#### Identification of chronic HypoR of MCAs/o patients

**Figure 2:**
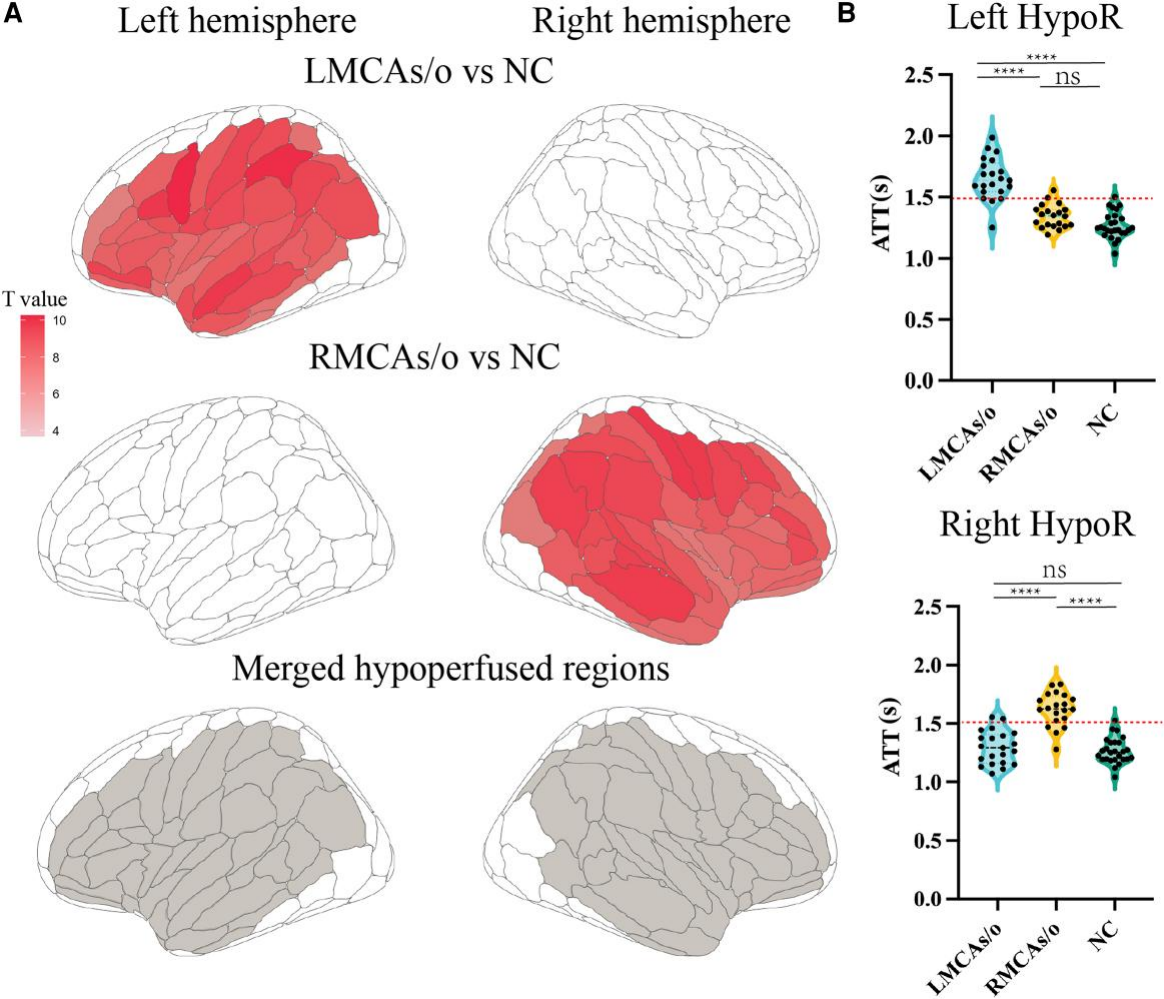
**Identification of chronic hypoperfusion area in MCAs/o patients based on ATT difference.** (**A**) The difference of ATT amongst MCAs/o patients and NC, with a merged hypoperfusion area shown below; (**B**) mean ATT within the HypoR (*n*: LMCAs/o = 21, RMCAs/o = 20, NC = 26, ns, no significant difference). Top subpanel: one-way ANOVA, *F* = 56.65, *P* < 0.0001; Bonferroni *post hoc* (*t*, adjusted *P*): LMCAs/o versus RMCAs/o *t* = 7.747, *P* < 0.0001; LMCAs/o versus NC *t* = 10.26, *P* < 0.0001; RMCAs/o versus NC *t* = 1.986, *P* = 0.1541. Bottom subpanel: one-way ANOVA, *F* = 49.45, *P* < 0.0001; Bonferroni *post hoc* (*t*, adjusted *P*): LMCAs/o versus RMCAs/o *t* = 8.072, *P* < 0.0001; LMCAs/o versus NC *t* = 0.7707, *P* > 0.9999; RMCAs/o versus NC *t* = 9.239, *P* < 0.0001. Note. ATT, arterial transit time; MCAs/o, middle cerebral artery stenosis/occlusion; NC, normal controls; HypoR, hypoperfusion regions.

Hemispheric cerebral cortex perfusion variable analysis showed that MCAs/o patients had significantly increased ATT in the stenotic hemisphere compared to NC, while CBF showed no significant changes (Supplementary Table 2). Further analysis based on BNA revealed that left MCAs/o patients had increased ATT in 41 ROIs (all located in the left MCA perfusion area, FDR-*P* < 0.0001, Fig. 2A), while right MCAs/o patients had increased ATT in 44 ROIs (all located in the right MCA perfusion area, FDR-*P* < 0.0001, Fig. 2A). In the ROI-based CBF analysis, there were no significantly altered ROIs in MCAs/o patients after FDR correction. These findings suggest that in the perfusion area of the stenotic MCA, perfusion impairment is evident, characterized by increased ATT, while CBF is not significantly reduced. Based on the group comparison result and considering the homology of BNA ROIs in the left and right hemispheres, we selected 41 ROIs with significantly increased ATT in the stenotic hemisphere MCAs/o patients for subsequent analysis (Fig. 2A, Supplementary Table 3). The average ATT in the stenotic MCA side of patients was also significantly higher than NC, exceeding 1.5 s (*P* < 0.0001, Fig. 2B). The mean CBF within the HypoR did not show significant intergroup differences (Supplementary Fig. 1A). However, correlation analysis between the mean ATT and CBF of the HypoR revealed a significant negative correlation (Supplementary Fig. 1B). The receiver operating characteristic (ROC) analysis showed that ATT significantly outperformed CBF (Supplementary Fig. 1C). The above results collectively indicated that both ATT and CBF appear to jointly reflect the hypoperfusion state, with ATT providing a more sensitive measure of hypoperfusion.

### Hypoperfusion-induced brain function alternation

**Figure 3:**
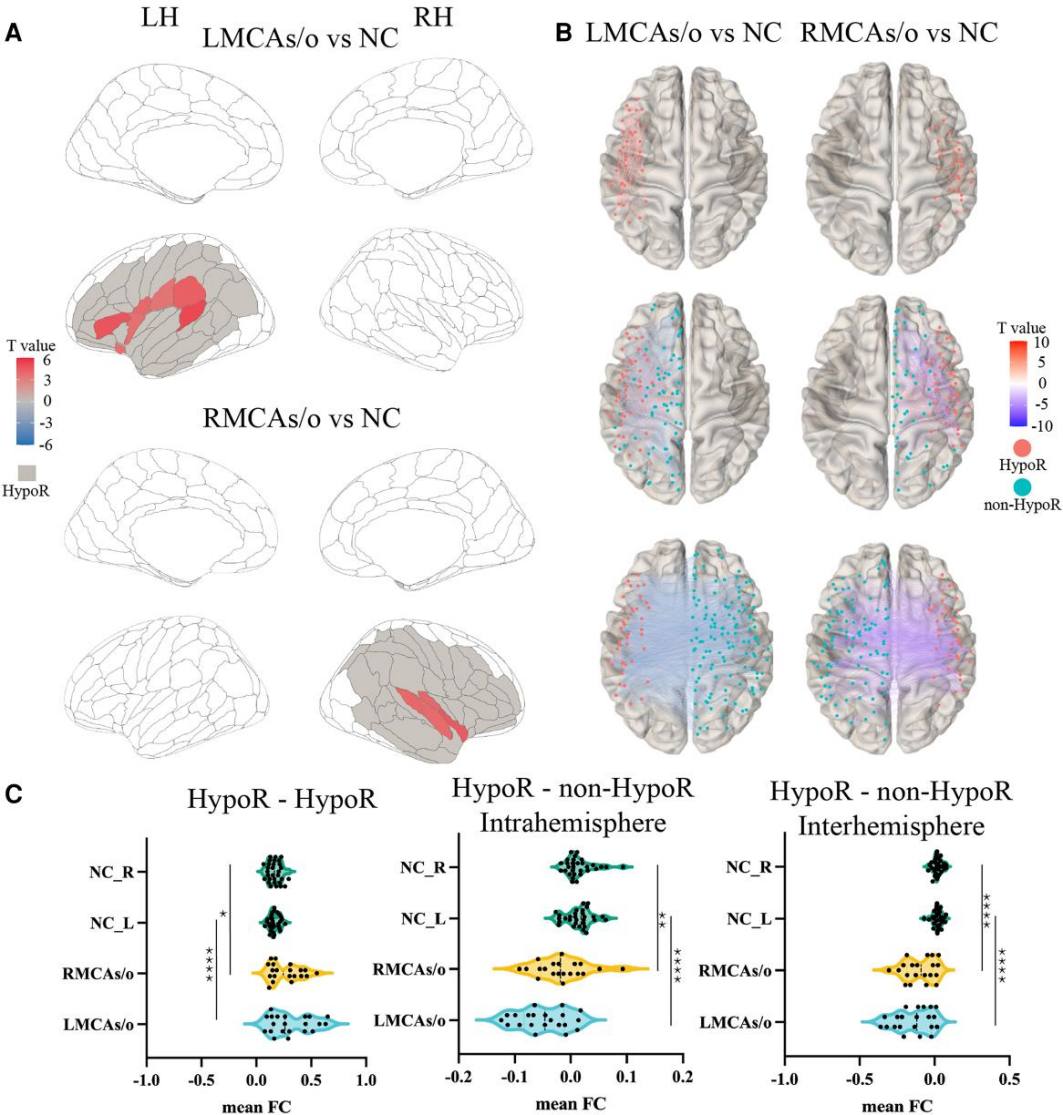
**Difference of functional measures amongst unilateral MCAs/o and NC groups.** (**A**) The difference of ReHo amongst MCAs/o patients and NC groups; (**B**) the difference of connectivity between the ROIs within the HypoR and both internal and external ROIs of the HypoR; (**C**) the difference of mean connectivity within the hypoperfused area amongst MCAs/o patients and NC groups (one-way ANOVA with Bonferroni *post hoc* comparisons; *n:* LMCAs/o = 21, RMCAs/o = 20, NC = 30). Left (HypoR–HypoR): ANOVA *F* = 11.28, *P* < 0.0001; *post hoc* (*t*, adjusted *P*): LMCAs/o versus NC_L *t* = 4.822, *P* < 0.0001; RMCAs/o versus NC_R *t* = 3.121, *P* = 0.0142. Middle (intrahemispheric HypoR–non-HypoR): ANOVA *F* = 19.92, *P* < 0.0001; *post hoc* (*t*, adjusted *P*): LMCAs/o versus NC_L *t* = 6.666, *P* < 0.0001; RMCAs/o versus NC_R *t* = 3.270, *P* = 0.0089. Right (interhemispheric HypoR–non-HypoR): ANOVA *F* = 31.10, *P* < 0.0001; *post hoc* (*t*, adjusted *P*): LMCAs/o versus NC_L *t* = 7.745, *P* < 0.0001; RMCAs/o versus NC_R *t* = 5.643, *P* < 0.0001. Note. (i) The HypoR refers to the ROIs defined in the previous ‘Arterial serial labelling preprocess and determination of hypoperfusion areas’ section; (ii) all labelled ROIs and functional connections in the figure have an FDR-*P* < 0.05; (iii) HypoR, hypoperfused regions; ReHo, regional homogeneity.

We first investigated local neural activity consistency in patients with MCAs/o by analysing the ReHo changes. Compared with the NC group, the left MCAs/o group had significant enhanced ReHo in seven brain regions which were all from left hemisphere (Fig. 3A**, FDR-corrected *P***  **<**  **0.05**). The right MCAs/o group had significant enhanced ReHo in four brain regions, which were all from right hemisphere (Fig. 3A**, FDR-corrected *P***  **<**  **0.05**). The MCAs/o group showed no significant decrease in ReHo values compared to the NC, and the increased areas were all located in the hypoperfusion area, suggesting that chronic hypoperfusion leads to compensatory increases in local neural activity consistency within the affected region.

To explore the impact of hypoperfusion on functional interactions between HypoR and other brain regions, we analysed the changes in connectivity between the ROIs within the HypoR and both internal and external ROIs of the HypoR. Compared to NC, the quantity of decreased functional connections outweighed the number of increased functional connections in the MCAs/o group, and it was also noticeable that the connections between ROIs within the chronic hypoperfusion region primarily increased, while the connections between ROIs within the chronic hypoperfusion region and ROIs outside the hypoperfusion region primarily decreased (Fig. 3B, Fig. 3C). We also compared the changes in average connectivity across major functional networks between MCAs/o patients and NC. The results revealed impairments in functional networks, including the sensorimotor network and the frontoparietal network, in MCAs/o patients (Supplementary Fig. 2A).

### Individualized quantitative measurement of hypoperfusion- related functional abnormalities

We systematically analysed HFAi using machine-learning-based feature selection and classification techniques. The analysis included indicators reflecting local and distant functional activities within the HypoR, specifically local ReHo (41 features) and intra- and interconnectivity of the HypoR (9225 features). After feature selection based on Lasso, a total of 18 functional features were retained for further analysis.

**Figure 4:**
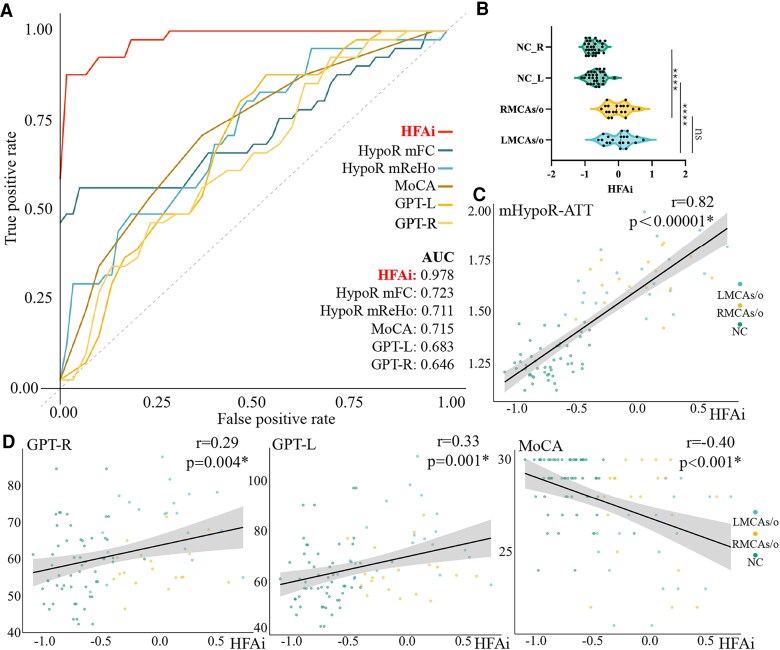
**Analysis of HFAi).** (**A**) ROC curves for HFAi and various measures; (**B**) the difference of HFAi amongst MCAs/o patients and NC groups (one-way ANOVA with Bonferroni *post hoc* comparisons; *n:* LMCAs/o = 21, RMCAs/o = 20, NC = 30, ns, no significant difference). ANOVA: *F* = 58.61, *P* < 0.0001; *post hoc* (*t*, adjusted *P*): LMCAs/o versus NC_L *t* = 9.251, *P* < 0.0001; RMCAs/o versus NC_R *t* = 9.401, *P* < 0.0001; LMCAs/o versus RMCAs/o *t* = 0.6168, *P* > 0.9999; (**C**) the relationship between HFAi and ATT with significant correlations (*P* < 0.00001); (**D**) the relationship between HFAi and cognitive test scores (MoCA, dominant/non-dominant hand GPT) with significant correlations (*P* < 0.01). Note. All correlation coefficients (*r*) and *P*-values are shown in the panels. HFAi, hypoperfusion-functional abnormality index; mHypoR-ATT, mean arterial transit time of the hypoperfused regions; HypoR-mFC, mean functional connection within the hypoperfused regions; HypoR-mReHo, mean ReHo within the hypoperfused regions; MoCA, Montreal cognitive assessment; NC, normal controls; MCAs/o, middle cerebral artery stenosis/occlusion; GPT, grooved pegboard test; AUC, area under the curve; ReHo, regional homogeneity.

A logistic regression model was applied for the calculation of HFAi using the reduced feature set. Left MCAs/o and right MCAs/o patients exhibited significantly different HFAi compared to NC (*P* < 0.0001, Fig. 4B). We then compared the ability of HFAi to detect abnormalities in MCAs/o patients and found that HFAi had a higher area under the ROC curve (AUC = 0.978) compared to MoCA (AUC = 0.715), dominant hand GPT (AUC = 0.646), non-dominant hand GPT (AUC = 0.683), average FC within the hypoperfused region (HypoR mFC, AUC = 0.723) and average ReHo in the hypoperfused region (HypoR mReHo, AUC = 0.711) (Fig. 4A). HFAi demonstrated a significantly greater ability to detect functional abnormalities in MCAs/o patients compared to the average connectivity of major functional networks (Supplementary Fig. 2B). Interestingly, we found that the HFAi of left MCAs/o showed a statistically non-significant increase compared to right MCAs/o (*P* = 0.64), indicating that the degree of functional changes caused by left or right MCA stenosis is similar.

Further research explored the relationship between HFAi, perfusion indicators and cognitive performance. We found that HFAi was significantly correlated with the average ATT in the hypoperfused region (mHypoR-ATT) of MCAs/o patients (*r* = 0.82, *P* < 0.00001, Fig. 4C). Interestingly, CBF also showed a significant negative correlation with HFAi (*r* = −0.29, *P* = 0.005, Supplementary Fig. 1D), which supports the notion that HFAi effectively captures the functional impact of hypoperfusion, with lower CBF reflecting greater neural dysfunction as represented by higher HFAi values. Additionally, we found significant correlations between HFAi and cognitive scores on the dominant hand GPT (GPT-R, *r* = 0.29, *P* = 0.004, Fig. 4D) and the non-dominant hand GPT (GPT-L, *r* = 0.33, *P* = 0.001, Fig. 4D), as well as significant correlations with MoCA (*r* = −0.40, *P* < 0.001, Fig. 4D).

### External validation of quantified hypoperfusion-related functional abnormalities

We validated the robustness of hypoperfusion-induced neural dysfunction using an independent MCAs/o cohort consisting of 11 left MCAs/o patients, 9 right MCAs/o patients and 18 healthy controls (Table 2). To ensure the validation's effectiveness, the MRI scans of these participants were conducted on a different machine from that used for the training/testing cohort.

**Table 2:** Demographic characteristic and neuropsychological assessment performance of the patients and controls in validation cohort

	LMCAs/o patients (*n* = 11)	RMCAs/o patients (*n* = 9)	Normal control (*n* = 18)
**Age (year) (mean** **±** **SD)**	48.09 ± 10.39	53 ± 11.05	60.33 ± 7.84
**Male, No. (%)**	3 (27.3%)	4 (44%)	11 (61.1%)
**Education level (mean** **±** **SD)**	10 ± 5.59	11.44 ± 3.28	10.89 ± 4.44
**MMSE (median [IQR])**	29 [28–30]	28 [28–29]	28 [27–29]
**MOCA (median [IQR])**	27 [26–29]	27 [26–28]	27 [26–28.75]
**Paired associate word learning test (median [IQR])**	8.5 [7–10.5]	9 [8–12]	9.25 [8–9.88]
**Rey complex figure copy (median [IQR])**	36 [35.5–36]	36 [36–36]	36 [34–36]
**Rey complex figure recall (median [IQR])**	20 [17.5–24.75]	21 [20–26.5]	18.25 [14.62–23.75]
**Grooved pegboard (dominant hand, median [IQR])**	67.06 [58.88–73.6]	63 [60.64–66]	63.12 [59.33–66.17]
**Grooved pegboard (non-dominant hand, median [IQR])**	66 [63.43–75.75]	72.11 [68.33–86.33]	69.31 [58.81–72.63]

**Figure 5:**
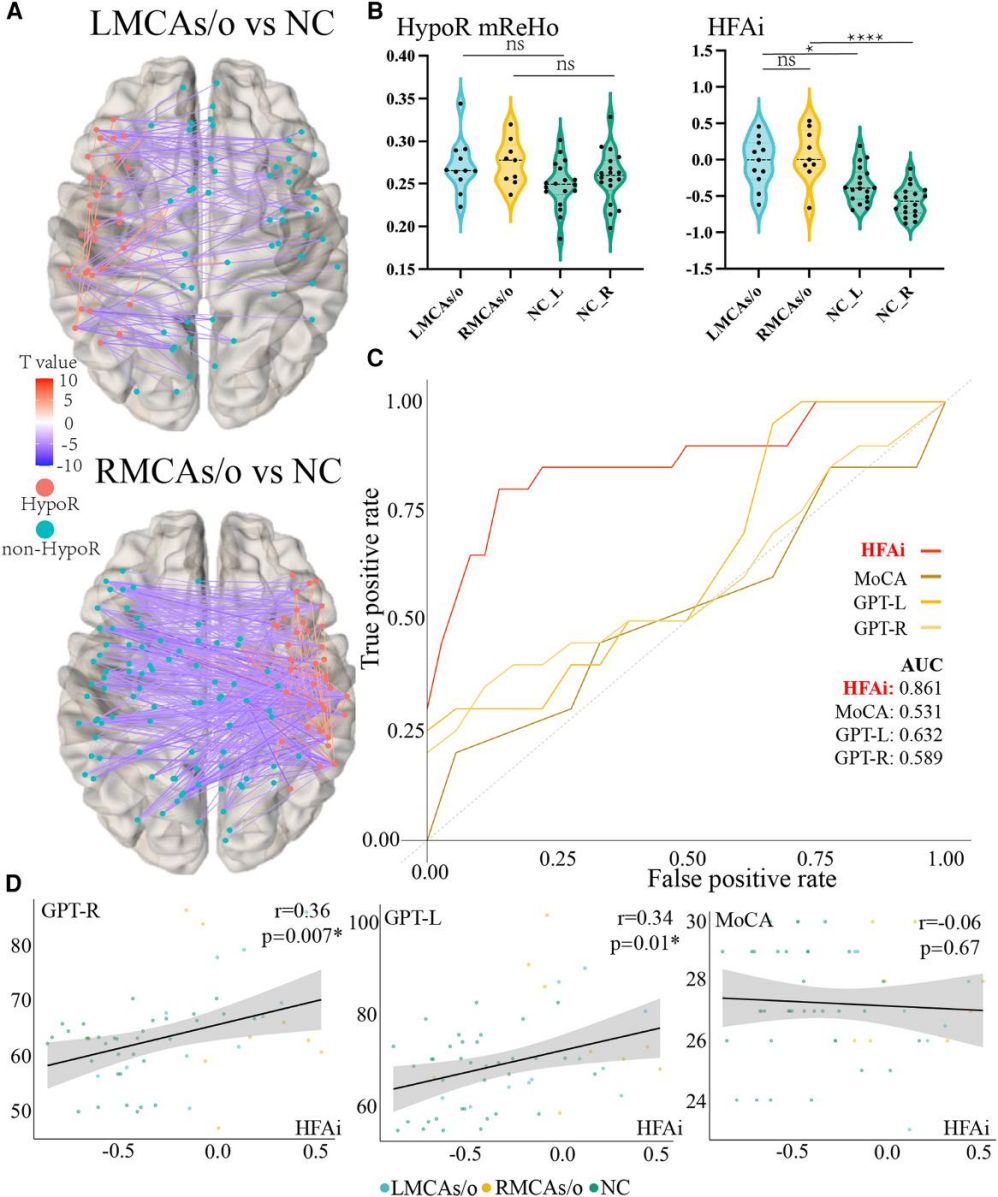
**Validation of HFAi in independent validation cohort.** (**A**) The difference of connectivity between the ROIs within the HypoR and both internal and external ROIs of the HypoR in validation cohort; (**B**) the difference of mean ReHo (left) and HFAi (right) within the hypoperfused area amongst MCAs/o patients and NC groups in validation cohort (one-way ANOVA with Bonferroni *post hoc* comparisons; *n:* LMCAs/o = 11, RMCAs/o = 9, NC = 18, ns, no significant difference). ReHo: ANOVA *F* = 2.113, *P* = 0.1098; *post hoc* (*t*, adjusted *P*): LMCAs/o versus NC_L *t* = 1.955, *P* = 0.3356; RMCAs/o versus NC_R *t* = 1.352, *P* > 0.9999; LMCAs/o versus RMCAs/o *t* = 0.2786, *P* > 0.9999; HFAi: ANOVA *F* = 14.21, *P* < 0.0001; *post hoc* (*t*, adjusted *P*): LMCAs/o versus NC_L *t* = 2.935, *P* = 0.0297; RMCAs/o versus NC_R *t* = 5.563, *P* < 0.0001; LMCAs/o versus RMCAs/o *t* = 0.7774, *P* > 0.9999; (**C**) ROC curves for HFAi and various measures in validation cohort; (**D**) the relationship between HFAi and cognitive test scores in validation cohort (MoCA, dominant/non-dominant hand GPT). Note. All correlation coefficients (*r*) and *P*-values are shown in the panels. HFAi, hypoperfusion-functional abnormality index; HypoR-mReHo, mean ReHo within the hypoperfused regions; MoCA, Montreal cognitive assessment; NC, normal controls; MCAs/o, middle cerebral artery stenosis/occlusion; GPT, grooved pegboard test; AUC, area under the curve; ROI, region of interest; ReHo, regional homogeneity.

We found highly reproducible patterns in the independent validation dataset, with increased connections between ROIs within the chronic hypoperfusion region and decreased connections between ROIs within the chronic hypoperfusion region and ROIs outside the hypoperfusion region (Fig. 5A). Additionally, MCAs/o patients showed a non-significant increase in ReHo within the HypoR compared to NC (Fig. 5B). HFAi showed significant inter-group differences between MCAs/o patients and NC (*P* < 0.05, Fig. 5B) and maintained excellent classification performance with an AUC of 0.861, compared to other cognition-based indicators (Fig. 5C). Additionally, HFAi was significantly correlated with the dominant hand GPT (*r* = 0.36, *P* = 0.007, Fig. 5D) and the non-dominant hand GPT (*r* = 0.34, *P* = 0.01, Fig. 5D), but no significant correlation was observed between HFAi and MoCA scores (*r* = −0.06, *P* = 0.67, Fig. 5D).

## Discussion

In this study, we systematically evaluated the impact of chronic hypoperfusion on regional neural function in patients with unilateral asymptomatic MCAs/o. We identified a distinct pattern of functional reorganization characterized by increased local functional consistency and connectivity within HypoR, alongside decreased connectivity between these regions and external areas. These abnormalities were quantified by the HFAi, which reflects heterogeneous neurocognitive impairments and was validated in an independent cohort.

The observed functional reorganization, i.e. increased local synchronization but reduced global integration, suggests a maladaptive response to chronic hypoperfusion.^34^ While heightened ReHo indicates local neural circuit synchronization, it likely represents inefficient compensation rather than improved function. A similar phenomenon was seen in other pathological conditions such as stroke and schizophrenia, where localized hyper-synchronization is associated with impaired global efficiency, suggesting a shift from integrated network function to more isolated processing and potentially leading to cognitive impairments.^34-37^ The altered FC patterns within and beyond the affected regions, showing reduced long-range connectivity, may also lead to functional impairments.^38-41^A comparable trend has also been observed in chronic hypoperfusion due to asymptomatic carotid artery stenosis, where reduced connectivity between the affected hemisphere and contralateral homologous regions has been reported,^38^ though variability in hypoperfusion severity and collateral circulation complicates direct comparisons.^39^

The development of HFAi, integrating ReHo and intra-/interregional connectivity, represents a methodological advance. We reported HFAi as the signed distance to the logistic regression decision boundary to provide a zero-centred, directionally interpretable index of hypoperfusion-related functional abnormality, which was widely used in neuroimaging studies and can quantify how far a patient’s biomarker pattern deviates from a normal reference and thus captures between-patient heterogeneity, supporting both the methodological feasibility and the interpretability of this choice.^21,32,42,43^ In both the discovery and validation cohorts. HFAi had significant correlations with specific cognitive deficits, particularly fine motor coordination as assessed by dominant and non-dominant hand GPT, likely reflecting the involvement of HypoR connected to the supplementary motor areas and presupplementary motor areas.^44^ Although the correlation between HFAi and MoCA was not replicated in the validation cohort, this discrepancy may be attributed to the relatively mild global cognitive impairment in MCAs/o patients and the higher baseline MoCA performance in the validation cohort. Nevertheless, the consistent negative trend across the study suggests HFAi’s potential to predict cumulative cognitive impairments. We did not observe other cognitive measures that have significant correlations with HFAi in the validation cohort. This may be explained by the fact that complex tasks (e.g. working memory, executive function, visual-spatial reasoning) rely on networks spanning multiple vascular territories, which are less susceptible to localized hypoperfusion.^45^ However, the negative correlation trends between HFAi and various cognitive scales in the discovery cohort suggest potential associations with broader cognitive domains, reinforcing the utility of HFAi as a marker of hypoperfusion-related dysfunction.

We also observed MCAs/o patients exhibited significantly prolonged ATT in HypoR despite preserved CBF, highlighting ATT’s sensitivity to subtle perfusion abnormalities.^46,47^ This suggests that chronic hypoperfusion can occur with only mild CBF reductions, as seen in asymptomatic carotid artery stenosis.^48^ The negative correlation between HFAi and CBF further underscores HFAi’s ability to capture functional impairments even in the absence of significant CBF reductions, complementing ATT’s role in detecting early hypoperfusion.

This study has several limitations. First, the focus on unilateral asymptomatic MCAs/o, while providing a well-defined model, may limit generalizability to more heterogeneous ICAS populations. Second, the cross-sectional design precludes investigation of dynamic changes in HFAi over time, warranting longitudinal studies. Finally, although our sample size is larger than that of similar studies and we used an independent validation cohort to verify the robustness of HFAi, further multi-modal imaging studies are still needed to confirm our findings.

## Conclusion

We characterized the specific patterns of functional abnormalities in patients with unilateral asymptomatic MCAs/o, which are closely linked to neurobehavioral alterations and can be objectively quantified by HFAi, offering a promising tool for personalized brain health evaluation.

## Supplementary Material

fcaf393_Supplementary_Data

## Data Availability

The data that support the findings of this study are available from the corresponding author upon reasonable request. Analysis scripts used in the manuscript are available at: https://github.com/yinxi-zou/MCAso-hypoperfusion-functional-abnormality-code.
