# Supplementary material for: Hypoperfusion-related functional abnormalities of middle cerebral artery stenotic-occlusive disease
Source: Brain Commun. 2025 Oct 14;7(5):fcaf393. doi: 10.1093/braincomms/fcaf393 (PMC12560163; doi:10.1093/braincomms/fcaf393)
Supplement: fcaf393_Supplementary_Data [file fcaf393_supplementary_data.docx]

## Supplementary Data

## Data S1 Neuroimaging protocol of the discovery and validation cohort

MR imaging of both the discovery and validation cohorts was performed on 3T MRI systems, though different MRI scanner models were used. The discovery cohort was scanned on a 3T Discovery systems (GE Healthcare), while the validation cohort was scanned on a MAGNETOM Skyra 3T MR scanner (Siemens, Erlangen, Germany). Despite the difference in scanner models, the scanning parameters for 3D T1-weighted imaging and resting-state functional MRI (rs-fMRI) sequences were identical between the two cohorts. Detailed parameters for both cohorts are provided in the Supplementary Material. All images were collected within one week following the neuropsychological testing. Participants were instructed to keep their eyes closed and their heads still during the scan, while avoiding falling asleep or engaging in cognitive processes. Foam padding was used to minimize head motion.

**1. Resting-state functional MRI (rsfMRI) parameters**

rsfMRI was acquired with the following parameters: repetition time (TR) = 2500 ms, echo time (TE) = 30 ms, slice thickness = 3.0 mm, voxel size = 3.0 x 3.0 x 3.0 mm³, flip angle = 90°, and field of view (FOV) = 240 x 240 mm². Each session acquired 200 functional volumes. All slices were scanned parallel to the anterior-posterior commissural line.

**2. 3D T1-weighted MRI parameters**

A 3D T1-weighted multi-echo magnetization-prepared rapid gradient-echo sequence was performed to acquire high-resolution images of brain structure. The sequence parameters were as follows: repetition time (TR) = 2300 ms, first echo time (TE1) = 1.69 ms, second echo time (TE2) = 3.3 ms, third echo time (TE3) = 4.91 ms, fourth echo time (TE4) = 6.52 ms, inversion time (TI) = 900 ms, flip angle = 9°, bandwidth = 890 Hz, echo spacing = 9 ms, and field of view (FOV) = 256 x 240 mm².

**3. Pseudocontinuous arterial spin labeling (pCASL) imaging parameters**

To obtain the participants’ cerebral blood flow (CBF) and arterial transit time (ATT) status for quantifying the chronic hypoperfusion areas in discovery cohort, a pCASL sequence was applied with the following parameters: A Hadamard-encoded multi-delay ASL was utilized with 7 post labeling delays (1.00, 1.22, 1.48, 1.78, 2.15, 2.63, 3.32 sec) and a long labeling block (3.5 sec) was divided into seven subboli (0.22, 0.26, 0.30, 0.37, 0.48, 0.68, 1.18 sec). Additional parameters were as follows: TR/TE = 6073/11.6 ms, FOV = 240 × 240 mm, in-plane spiral arms number 4, points per arms = 512, slice thickness = 4 mm, 40 slices, acquisition time = 4 min 6s. In addition, TOF-MRA images were collected to affirm that all patients only had stenosis in the MCA.

## Supplementary Figures

## sFigure 1 Analysis of cerebral blood flow in hypoperfused regions


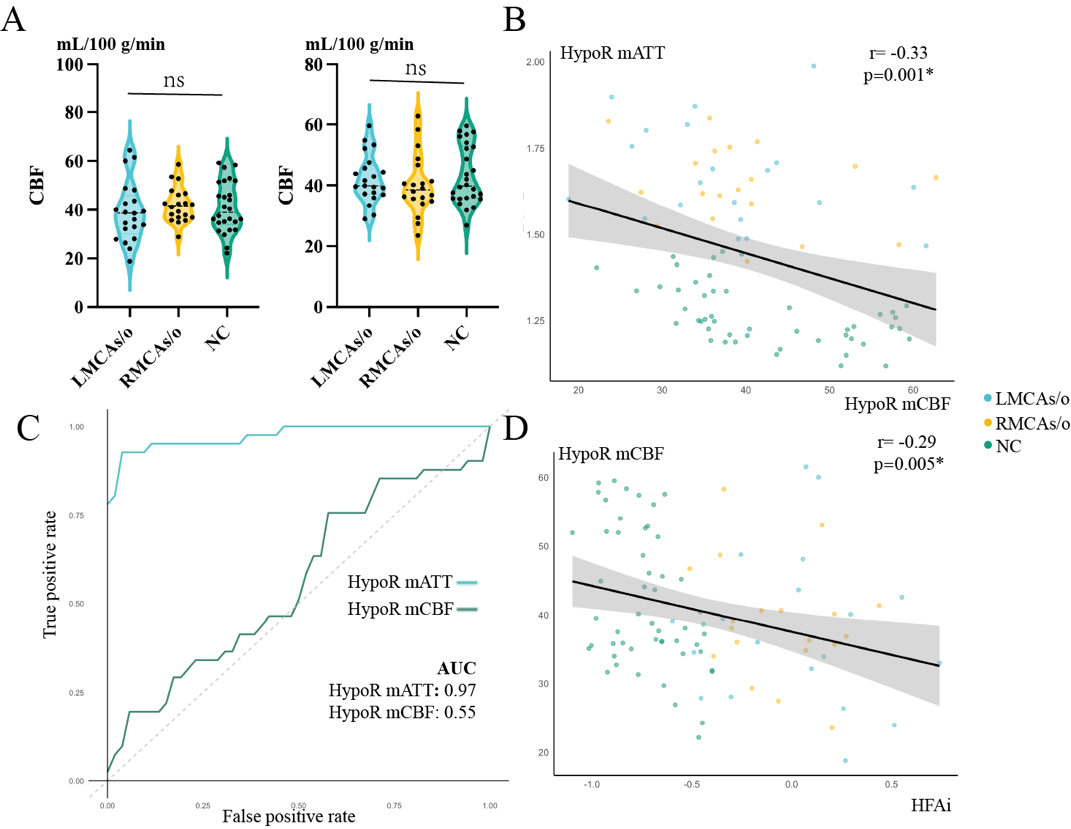


**A,** the difference in the average CBF within the hypoperfused region between MCAs/o patients and normal controls (one-way ANOVA with Bonferroni post-hoc comparisons; *n:* LMCAs/o=21, RMCAs/o=20, NC=26, ns, no significant difference). Left subpanel (left HypoR): ANOVA F=0.4413, P=0.6451; post-hoc (t, adjusted P): LMCAs/o vs NC t=0.6535, P>0.9999; LMCAs/o vs RMCAs/o t=0.9162, P>0.9999; RMCAs/o vs NC t=0.3178, P>0.9999; Right subpanel (right HypoR): ANOVA F=0.6297, P=0.5360; post-hoc (t, adjusted P): LMCAs/o vs NC t=0.4136, P>0.9999; LMCAs/o vs RMCAs/o t=0.6766, P>0.9999; RMCAs/o vs NC t=1.119, P=0.8023; **B,** the relationship between average CBF and ATT within the hypoperfused region; **C,** receiver operating characteristic (ROC) curves for average CBF and ATT within the hypoperfused region; **D,** the relationship between HFAi and average CBF within the hypoperfused region.

Note. All Pearson correlation coefficients (r) and p values are shown in the panels; MCAs/o, asymptomatic middle cerebral artery stenotic-occlusive disease; HypoR mATT, average arterial transit time within the hypoperfused region; HypoR mCBF, average cerebral blood flow within the hypoperfused region; HFAi, hypoperfusion-functional abnormality index.

## sFigure 2 Correlation analysis between HFAi and other cognitive scales in the discovery cohort


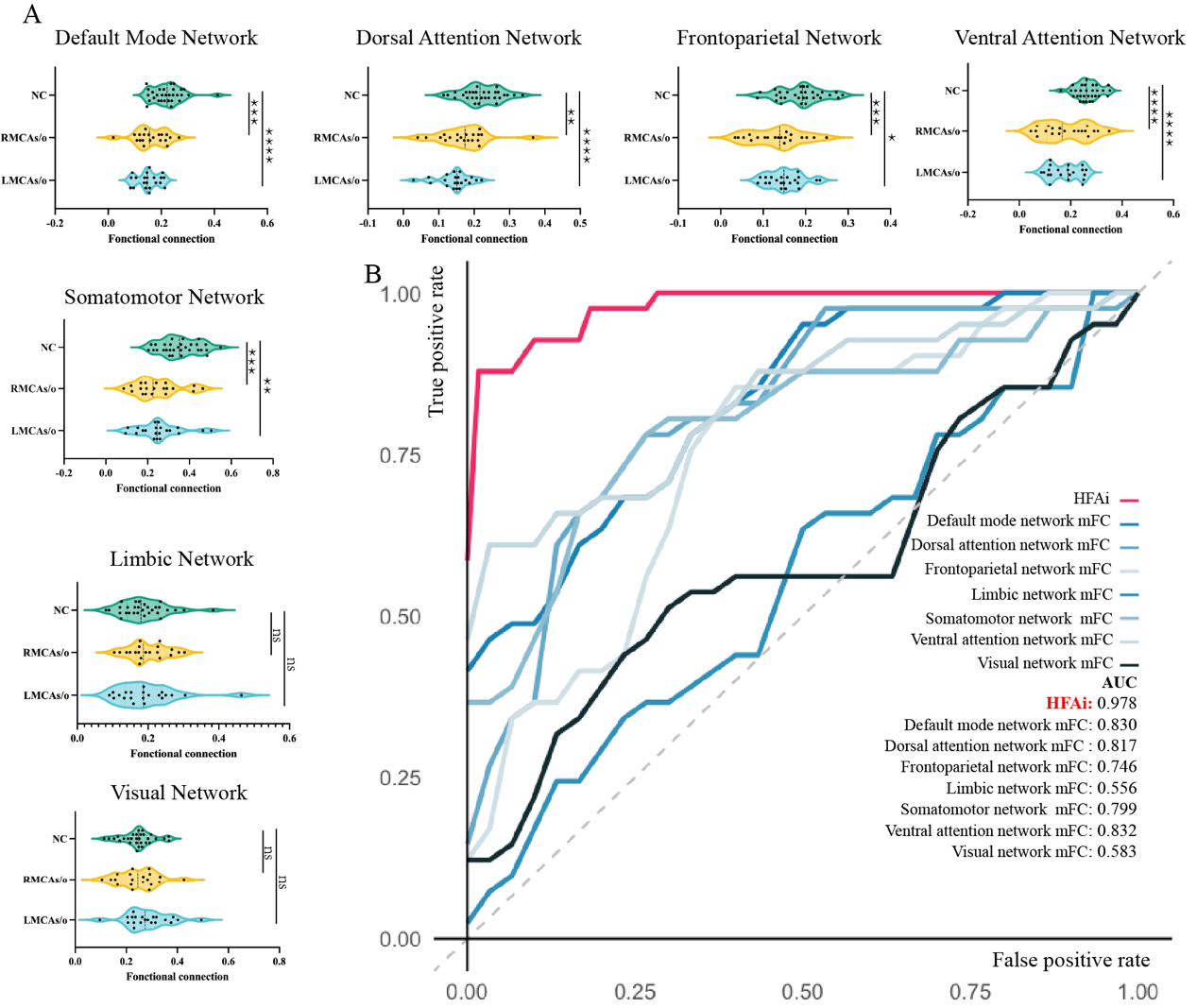


**A,** the average functional connectivity of main functional networks (default mode, frontoparietal, somatomotor, dorsal attention, ventral attention, limbic, visual network; (one-way ANOVA with Bonferroni post-hoc comparisons; *n:* LMCAs/o=21, RMCAs/o=20, NC=30, ns, no significant difference). Default mode network: ANOVA F=13.90, P<0.0001; post-hoc (adjusted P): LMCAs/o vs NC <0.0001; RMCAs/o vs NC 0.0005. Frontoparietal network: ANOVA F=8.112, P=0.0007; post-hoc (adjusted P): LMCAs/o vs NC 0.0370; RMCAs/o vs NC 0.0007. Somatomotor network: ANOVA F=10.00, P=0.0002; post-hoc (adjusted P): LMCAs/o vs NC 0.0019; RMCAs/o vs NC 0.0007. Dorsal attention network: ANOVA F=12.29, P<0.0001; post-hoc (adjusted P): LMCAs/o vs NC <0.0001; RMCAs/o vs NC 0.0074. Ventral attention network: ANOVA F=15.73, P<0.0001; post-hoc (adjusted P): LMCAs/o vs NC <0.0001; RMCAs/o vs NC <0.0001. Limbic network: ANOVA F=0.1884, P=0.8287; post-hoc (adjusted P): LMCAs/o vs NC >0.9999; RMCAs/o vs NC >0.9999. Visual network: ANOVA F=2.062, P=0.1351; post-hoc (adjusted P): LMCAs/o vs NC 0.2026; RMCAs/o vs NC >0.9999. **B,** receiver operating characteristic (ROC) curves for HFAi and average functional connectivity of main functional networks.

Note. MCAs/o, asymptomatic middle cerebral artery stenotic-occlusive disease; HFAi, hypoperfusion-functional abnormality index.

## Supplementary Tables

## sTable 1 Basic neuroimaging data of the patients and controls

|  | | **LMCAs/o patients**  **(n=21)** | **RMCAs/o patients**  **(n=20)** | **Normal control**  **(n=30)** | **P value** |
| --- | --- | --- | --- | --- | --- |
| *******Lacunar scale (0:1:2:3)** | 18:2:1:0 | | 19:1:0:0 | 30:0:0:0 | 0.25 |
| **Fazekas scale (0:1:2:3)** | 6:14:1:0 | | 11:7:2:0 | 29:1:0:0 | ＜0.001 |
| ****No/mild white matter hyperintensities, No. (%)** | 20(95.2%) | | 18(90%) | 30(100%) | 0.23 |
| **Whole brain atrophy, No. (%)** | 3(14.3%) | | 0(0.00%) | 4(13.3%) | 0.22 |
| **Temporal lobe atrophy, No. (%)** | 2(9.5%) | | 0(0.00%) | 2(6.7%) | 0.40 |

Note. *Lacunar scale, scale for the number of lacunar in the subjects (0 = no lacunar; 1 = 1-2 lacunars; 2 = 3-5 lacunars; 3 = >5 lacunars;); **No/mild white matter hyperintensities, subjects with Fazekas scale 0-1.

## sTable 2 Hemisphere cerebral cortex perfusion parameters of the patients and controls

| **Perfusion parameters** | **LMCAs/o patients**  **(n=21)** | **RMCAs/o patients**  **(n=20)** | **Normal control**  **(n=26)** | **P value**  **(LMCAs/o vs NC)** | **P value**  **(RMCAs/o vs NC)** |
| --- | --- | --- | --- | --- | --- |
| **Arterial transit time (s)** |  |  |  |  |  |
| **Left cerebral cortex (Mean±SD)** | 1.53±0.12 | 1.45±0.09 | 1.38±0.10 | <0.0001 | 0.03 |
| **Right cerebral cortex (Mean±SD)** | 1.42±0.13 | 1.53±0.06 | 1.38±0.11 | 0.28 | <0.0001 |
| **Cerebral blood flow (mL/100 g/min)** |  |  |  |  |  |
| **Left cerebral cortex (Mean±SD)** | 42.8±7.5 | 40.9±5.4 | 41.2±7.4 | 0.47 | 0.89 |
| **Right cerebral cortex (Mean±SD)** | 41.7±6.3 | 43.5±5.7 | 42.6±7.6 | 0.68 | 0.64 |

## sTable 3 Chronic hypoperfusion ROIs in MCAs/o patients

| **Lobe** | **Gyrus** | **ROI** | **Anatomical and modified Cyto-architectonic descriptions** |
| --- | --- | --- | --- |
| Frontal Lobe | MFG, Middle Frontal Gyrus | MFG_L(R)_7_2 | IFJ, inferior frontal junction |
|  |  | MFG_L(R)_7_4 | A9/46v, ventral area 9/46 |
|  | IFG, Inferior Frontal Gyrus | IFG_L(R)_6_1 | A44d,dorsal area 44 |
|  |  | IFG_L(R)_6_2 | IFS, inferior frontal sulcus |
|  |  | IFG_L(R)_6_3 | A45c, caudal area 45 |
|  |  | IFG_L(R)_6_4 | A45r, rostral area 45 |
|  |  | IFG_L(R)_6_5 | A44op, opercular area 44 |
|  |  | IFG_L(R)_6_6 | A44v, ventral area 44 |
|  | OrG, Orbital Gyrus | OrG_L(R)_6_2 | A12/47o, orbital area 12/47 |
|  |  | OrG_L(R)_6_6 | A12/47l, lateral area 12/47 |
|  | PrG, Precentral Gyrus | PrG_L(R)_6_1 | A4hf, area 4(head and face region) |
|  |  | PrG_L(R)_6_5 | A4tl, area 4(tongue and larynx region) |
|  |  | PrG_L(R)_6_6 | A6cvl, caudal ventrolateral area 6 |
| Temporal Lobe | STG, Superior Temporal Gyrus | STG_L(R)_6_1 | A38m, medial area 38 |
|  |  | STG_L(R)_6_2 | A41/42, area 41/42 |
|  |  | STG_L(R)_6_3 | TE1.0 and TE1.2 |
|  |  | STG_L(R)_6_4 | A22c, caudal area 22 |
|  |  | STG_L(R)_6_5 | A38l, lateral area 38 |
|  |  | STG_L(R)_6_6 | A22r, rostral area 22 |
|  | MTG, Middle Temporal Gyrus | MTG_L(R)_4_1 | A21c, caudal area 21 |
|  |  | MTG_L(R)_4_2 | A21r, rostral area 21 |
|  |  | MTG_L(R)_4_3 | A37dl, dorsolateral area37 |
|  |  | MTG_L(R)_4_4 | aSTS, anterior superior temporal sulcus |
|  | ITG, Inferior Temporal Gyrus | ITG_L(R)_7_4 | A20il, intermediate lateral area 20 |
|  | pSTS, posterior Superior Temporal Sulcus | pSTS_L(R)_2_1 | rpSTS, rostroposterior superior temporal sulcus |
|  |  | pSTS_L(R)_2_2 | cpSTS, caudoposterior superior temporal sulcus |
| Parietal Lobe | SPL, Superior Parietal Lobule | SPL_L(R)_5_3 | A5l, lateral area 5 |
|  | IPL, Inferior Parietal Lobule | IPL_L(R)_6_2 | A39rd, rostrodorsal area 39(Hip3) |
|  |  | IPL_L(R)_6_3 | A40rd, rostrodorsal area 40(PFt) |
|  |  | IPL_L(R)_6_4 | A40c, caudal area 40(PFm) |
|  |  | IPL_L(R)_6_5 | A39rv, rostroventral area 39(PGa) |
|  |  | IPL_L(R)_6_6 | A40rv, rostroventral area 40(PFop) |
|  | PoG, Postcentral Gyrus | PoG_L(R)_4_1 | A1/2/3ulhf, area 1/2/3(upper limb, head and face region) |
|  |  | PoG_L(R)_4_2 | A1/2/3tonIa, area 1/2/3(tongue and larynx region) |
|  |  | PoG_L(R)_4_3 | A2, area 2 |
| Insular Lobe | INS, Insular Gyrus | INS_L(R)_6_1 | G, hypergranular insula |
|  |  | INS_L(R)_6_2 | vIa, ventral agranular insula |
|  |  | INS_L(R)_6_3 | dIa, dorsal agranular insula |
|  |  | INS_L(R)_6_4 | vId/vIg, ventral dysgranular and granular insula |
|  |  | INS_L(R)_6_5 | dIg, dorsal granular insula |
|  |  | INS_L(R)_6_6 | dId, dorsal dysgranular insula |

Note. Considering the homology of BNA ROIs in the left and right hemispheres, we selected 41 ROIs with significantly increased ATT in the stenotic hemisphere of both LMCAs/o and RMCAs/o patients as the hypoperfusion areas based on ATT difference for subsequent analysis
